# Defining Stage-Specific Activity of Potent New Inhibitors of Cryptosporidium parvum Growth *In Vitro*

**DOI:** 10.1128/mBio.00052-20

**Published:** 2020-03-03

**Authors:** Lisa J. Funkhouser-Jones, Soumya Ravindran, L. David Sibley

**Affiliations:** a Department of Molecular Microbiology, Washington University School of Medicine, St. Louis, Missouri, USA; Albert Einstein College of Medicine

**Keywords:** cryptosporidiosis, enteric pathogen, target identification, small-molecule inhibitors, intestinal stem cells, primary cell culture, mechanism of action

## Abstract

Cryptosporidium parvum and Cryptosporidium hominis have emerged as major enteric pathogens of infants in the developing world, in addition to their known importance in immunocompromised adults. Although there has been recent progress in identifying new small molecules that inhibit *Cryptosporidium* sp. growth *in vitro* or in animal models, we lack information about their mechanism of action, potency across the life cycle, and cidal versus static activities. Here, we explored four potent classes of compounds that include inhibitors that likely target phosphatidylinositol 4 kinase (PI4K), phenylalanine-tRNA synthetase (PheRS), and several potent inhibitors with unknown mechanisms of action. We utilized monoclonal antibodies and gene expression probes for staging life cycle development to define the timing of when inhibitors were active during the life cycle of Cryptosporidium parvum grown *in vitro*. These different classes of inhibitors targeted different stages of the life cycle, including compounds that blocked replication (PheRS inhibitors), prevented the segmentation of daughter cells and thus blocked egress (PI4K inhibitors), or affected sexual-stage development (a piperazine compound of unknown mechanism). Long-term cultivation of C. parvum in epithelial cell monolayers derived from intestinal stem cells was used to distinguish between cidal and static activities based on the ability of parasites to recover from treatment. Collectively, these approaches should aid in identifying mechanisms of action and for designing *in vivo* efficacy studies based on time-dependent concentrations needed to achieve cidal activity.

## Abstract (precis)

Currently, nitazoxanide is the only FDA-approved treatment for cryptosporidiosis; unfortunately, it is ineffective in immunocompromised patients, has varied efficacy in immunocompetent individuals, and is not approved in infants under 1 year of age. Identifying new inhibitors for the treatment of cryptosporidiosis requires standardized and quantifiable *in vitro* assays for assessing potency, selectivity, timing of activity, and reversibility. Here, we provide new protocols for defining which stages of the life cycle are susceptible to four highly active compound classes that likely inhibit different targets in the parasite. We also utilize a newly developed long-term culture system to define assays for monitoring reversibility as a means of defining cidal activity as a function of concentration and time of treatment. These assays should provide valuable *in vitro* parameters to establish conditions for efficacious *in vivo* treatment.

## INTRODUCTION

Cryptosporidiosis is a debilitating diarrheal disease in humans that is largely caused by two species, Cryptosporidium parvum, a zoonotic species acquired primarily from agricultural animals that can also transmit between humans, and the anthroponotic Cryptosporidium hominis species, which is almost exclusively transmitted from human to human (1). Infections are most severe in immunocompromised patients (2) and infants under age 2, particularly in developing countries (3). Unfortunately, the only FDA-approved drug for the treatment of cryptosporidiosis, nitazoxanide, is largely ineffective in the most susceptible patient populations and is not licensed for infants under 1 year of age (4, 5). The identification of new compounds that inhibit C. parvum is hampered by the difficulty of *in vitro* propagation combined with animal models limited to immunocompromised mice (C. parvum), gnotobiotic piglets (C. hominis), or calves (C. parvum) (6).

Recent efforts have leveraged high-throughput screening platforms and repurposing screens to identify new compounds with the potential for advancement to clinical trials. A high-throughput screen of almost 80,000 small molecules identified 12 anticryptosporidial compounds that inhibit growth in the submicromolar range, including clofazimine (7). Screening of the open-access Malaria Box of compounds available through the Medicine for Malaria Venture (https://www.mmv.org/) identified a piperazine-based inhibitor (i.e., MMV665917) that showed potent activity in a NOD SCID gamma immunocompromised mouse model of chronic cryptosporidiosis (8), in neonatal dairy calves (9), and in gnotobiotic piglets infected with C. hominis (10). Screening of a focused library of antimalarial compounds identified imidazopyrazine compounds as potent inhibitors of C. parvum growth (11). This class of imidazopyrazines inhibits phosphatidylinositol 4 kinase (PI4K) in Plasmodium falciparum (12), an activity that may explain its potent ability to control C. parvum growth *in vitro* and *in vivo*. Prior studies have identified benzoxaboroles that act on mRNA polyadenylation in P. falciparum (13), and genetic evidence supports a similar target in Toxoplasma gondii (14). Related benzoxaboroles are potent inhibitors of C. parvum growth in an *in vitro* model and calf model of cryptosporidiosis (15). Previous studies in P. falciparum have also highlighted the potency of bicyclic azetidines that inhibit parasite phenylalanine-tRNA synthetases (PheRS) (16), suggesting that these may also have broad-spectrum activity against other apicomplexans. Consistent with this prediction, recent studies indicate that bicyclic azetidines are also potent inhibitors of C. parvum growth *in vitro* (17).

The majority of studies that have identified new inhibitors have utilized microtiter plate-based growth assays that do not rely on knowledge of specific targets. To better understand their mode of action, it would be beneficial to develop assays that identify when compounds act across the life cycle and to define the minimum concentration and time required to achieve complete killing *in vitro*. Deconvolving the targets of activity within the life cycle has been a major focus of successful efforts to define new compounds that inhibit Plasmodium spp. (18). Limitations in culturing C. parvum *in vitro* have made it difficult to perform similar studies, although methods have recently been described for staging the activity of inhibitors in tumor cell lines, where partial development takes place (17).

*Cryptosporidium* spp. undergo their entire life cycle in a single host, consisting of several rounds of asexual amplification followed by sexual differentiation and fertilization to form an oocyst (19). C. parvum can be propagated for several rounds of asexual growth in a variety of tumor cell lines *in vitro*, and although it develops into gametocytes, it does not complete the sexual cycle to form oocysts. Thus, only short-term propagation is possible in these systems (20). Recent efforts have developed new platforms to alleviate this restriction and have led to organoid-based systems that use human stem cell-derived cultures to propagate C. parvum and to produce oocysts that are infectious to mice (21). However, this system requires microinjection of parasites and does not allow ready access for experimental manipulation. As an alternative system for long-term propagation of C. parvum, we have recently described a mouse enterocyte model that is based on the propagation of intestinal stem cells, followed by differentiation on two-dimensional (2D) transwell filters (22, 23). Removal of the liquid medium from the upper chamber to create an air-liquid interface (ALI) induces differentiation of intestinal cell lineages and favors the growth of C. parvum (22, 23). Importantly, the ALI culture system is amenable to adding compounds for defined intervals of treatment, and because transwells are grown in microtiter plates, the system can be scaled easily to evaluate multiple parallel cultures.

Here, we sought to examine several newly identified compounds that are potent inhibitors of C. parvum growth *in vitro*. We were interested in defining the window of development when inhibitors are active across the life cycle. We took advantage of the fact that the cycle is somewhat synchronous in HCT-8 adenocarcinoma cells, combined with newly defined antibodies (24) and gene probes for defining the stages of the life cycle, to profile when inhibitors show peak activity. We also used the long-term ALI cell culture to define time- and concentration-dependent conditions required for cidal activity. Together, these tools provide a defined set of reagents and assays for profiling compounds that inhibit C. parvum *in vitro* and help establish guidelines for achieving effective control *in vivo*.

## RESULTS

### Efficacy of selected anti-*Cryptosporidium* compounds *in vitro*.

**FIG 1:**
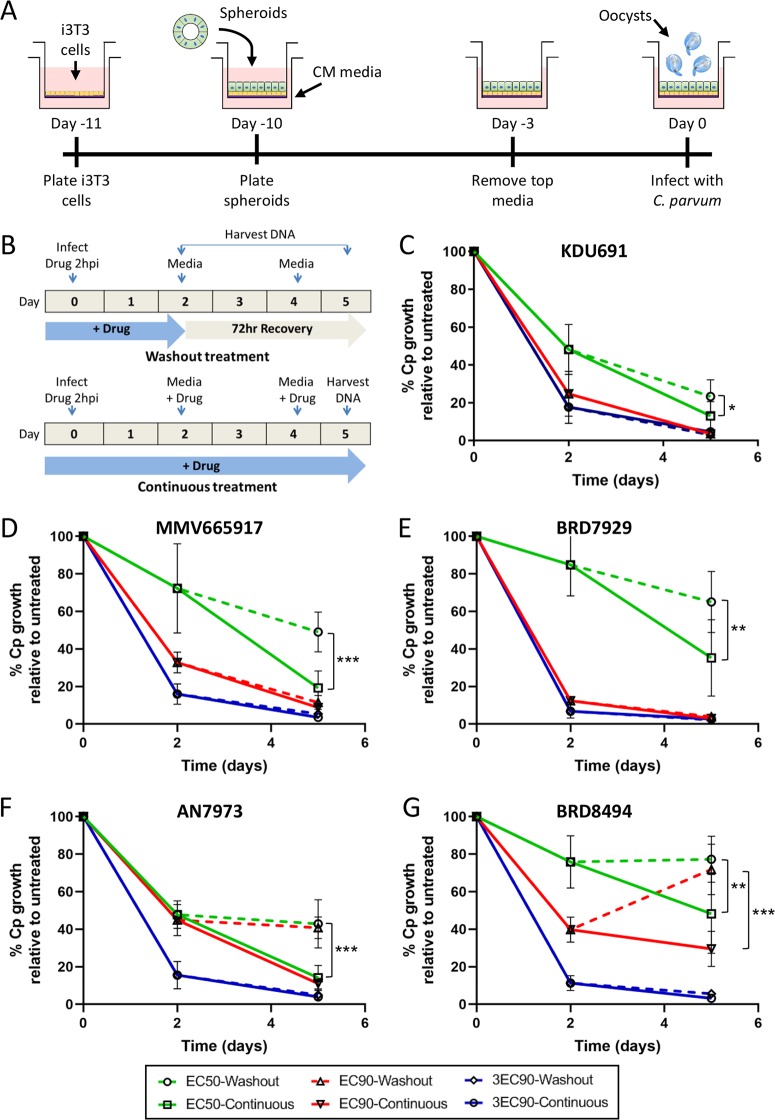
Testing time-dependent killing of C. parvum by compounds under air-liquid interface (ALI) culture conditions. (A) Mouse intestinal spheroids were trypsinized and plated on top of irradiated 3T3 (i3T3) feeder cells and Matrigel. The culture was grown in 50% conditioned medium (CM) containing 10 μM ROCK inhibitor. On day 7, medium from the top chamber of the transwell was removed to create the air-liquid interface. Samples were infected 3 days post-medium removal. (B) Compounds were added 2 hpi to transwell monolayers (top and bottom compartments). The transwells were treated with compound for 48 h (washout treatment) or for the entire length of the experiment (5 days) in parallel wells. DNA was harvested at 0, 2, and 5 days postinfection. (C to G) For KDU691 (C), MMV665917 (D), BRD7929 (E), AN7973 (F), and BRD8494 (G) washout experiments, C. parvum genome equivalents were quantified using qPCR and normalized to a no-compound control for each time point. Each compound was tested at its ALI EC_50_ (green), EC_90_ (red), and 3× the EC_90_ (blue), with parasite recovery after washout represented by dashed lines and continuous treatment represented by solid lines. Data represent the mean ± standard deviation (SD) for four separate replicate wells from two independent experiments. Statistical analysis was performed using two-way ANOVA corrected for multiple comparisons by Sidak’s method. *, *P < *0.05; **, *P < *0.01; ***, *P < *0.001.

We focused our study on four classes of potent anti-*Cryptosporidium* compounds identified in previous screening efforts, bicyclic azetidines BRD7929 and BRD8494, the imidazopyrazine KDU691, the benzoxaborole AN7973, and the piperazine MMV665917. To confirm their efficacy with our C. parvum strain, AUCP-1, we performed dose-curve assays in HCT-8 cells using a medium-throughput imaging assay (see Materials and Methods) and calculated their respective 50% and 90% effective concentration (EC_50_ and EC_90_, respectively) values from three independent experiments (Table 1). To determine whether the compounds would be effective under conditions that better mimic the parasite’s natural niche, we performed similar dose-response curves in an ALI system that allows long-term cultivation of C. parvum *in vitro* (22, 23). Briefly, mouse ileal stem cell spheroids were plated in transwells on top of an irradiated fibroblast feeder cell layer (Fig. 1A). Monolayers were cultured in conditioned medium (CM) (25–27) for 7 days, at which point the top medium was removed to form the air-liquid interface and promote differentiation of the monolayers. Oocysts were added to the monolayers 3 days after removal of the top medium, and serial dilutions of the compounds were added to the top and bottom chambers of the transwell. After 48 h of compound treatment, EC_50_ and EC_90_ values were calculated based on the number of C. parvum genome equivalents in each transwell as quantified by quantitative PCR (qPCR). Even though the times of treatment between the two assays differed (24 h for HCT-8 cells versus 48 h for ALI cultures), all compounds showed a <4-fold change in EC_50_ values between the two systems, except for nitazoxanide, which was ∼12-fold less potent against C. parvum in ALI than in HCT-8 cultures (Table 1).

**TABLE 1:** EC_50_ and EC_90_ values for compounds against C. parvum grown in HCT8 versus ALI cultures

Compound	EC_50_ (μM) in (mean ± SD):		Fold change^*c*^	EC_90_ (μM) in (mean ± SD):	
	HCT8 cells^*a*^	ALI cells^*b*^		HCT8 cells^*a*^	ALI cells^*a*^
Nitazoxanide	2.190 ± 0.378	25.940 ± 4.137	11.8	6.497 ± 2.632	49.250 ± 26.110
KDU691	0.053 ± 0.019	0.132 ± 0.022	2.5	0.164 ± 0.069	0.392 ± 0.130
BRD7929	0.033 ± 0.009	0.113 ± 0.015	3.4	0.225 ± 0.041	0.847 ± 0.264
BRD8494	0.011 ± 0.003	0.028 ± 0.002	2.5	0.081 ± 0.019	0.055 ± 0.020
AN7973	0.347 ± 0.129	0.633 ± 0.359	1.8	1.378 ± 0.500	0.778 ± 0.205
MMV665917	2.360 ± 0.643	3.607 ± 0.944	1.5	9.543 ± 3.568	7.540 ± 2.649

a *n* = 3; 9-point (pt) curve. Calculated as log(inhibitor) versus normalized response – variable slope. The assay is based on 24 h of growth.

b *n* = 2; 5-pt curve. Calculated as log(inhibitor) versus normalized response – variable slope. The assay is based on 48 h of growth.

c Fold change is defined as the ALI-EC_50_ divided by the HCT8-EC_50_.

### Reversibility of compound effects on C. parvum infection during long-term culture.

Although assays to test the cidal versus static properties of compounds against C. parvum in HCT-8 cells have been described (8), the lack of C. parvum growth after the first 72 h in transformed cell lines limits the ability of these assays to test parasite recovery after compound removal. Thus, we performed washout experiments in our long-term ALI culture system to determine whether treatment with compounds for 48 h was sufficient to kill C. parvum or if parasite growth would resume after compound removal during a 72-h recovery period (Fig. 1B). Each compound was tested at three different concentrations (EC_50,_ EC_90,_ and 3× the EC_90_ for ALI conditions; Table 1) to see whether recovery dynamics changed as the concentration of compound increased. Due to the much less potent activity of nitazoxanide in ALI culture (Table 1), it was not included in these washout assays.

**FIG 2:**
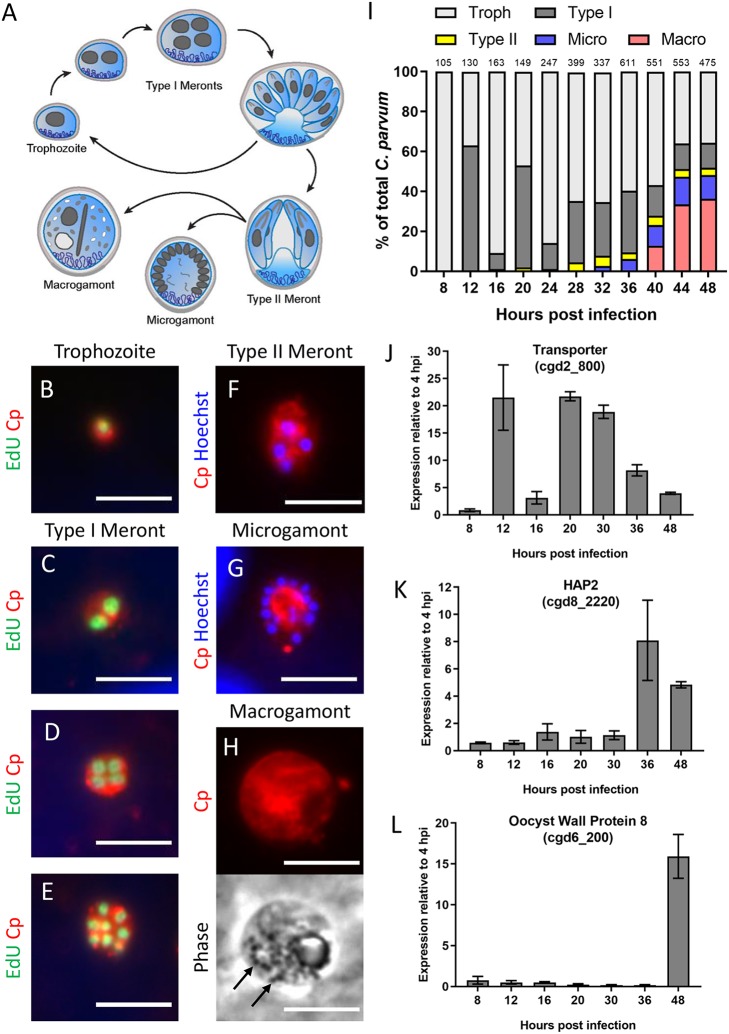
Characterization of C. parvum intracellular stages over the first 48 h of infection in HCT-8 cells. (A) Diagram of the C. parvum intracellular life cycle. Single-nucleus trophozoites replicate mitotically to form eight mature type I merozoites that egress and reinvade host cells. After at least two rounds of asexual replication, parasites divide into four mature type II meronts, which differentiate into one of two sexual life stages, macrogamonts or multinucleated microgamonts. (B to H) Immunostaining of parasite stages with rabbit polyclonal anti-C. parvum (Cp, red) and either EdU, a fluorescent thymidine analog that incorporates into replicating DNA (green), or the nuclear stain Hoechst (blue). Life cycle stages were distinguished as follows: trophozoites, with the presence of a single nuclei (B); type I meronts with the presence of two nuclei C), four EdU^+^ nuclei (D), or eight nuclei (E); type II meronts with four EdU^−^ nuclei (F); microgamonts with more than eight nuclei (G); and macrogamonts, with the presence of wall-forming bodies visible by phase contrast (black arrows) (H). Scale bars = 5 μm. (I) Abundance of each life cycle stage as a percentage of total C. parvum at the indicated hours postinfection (hpi). For each time point, EdU was added to the culture 2 h prior to fixation and antibody labeling, and the number of parasites at each stage was counted for 10 fields of view with a 100× oil objective. The number of total parasites counted per time point is shown above bar graph. Troph, trophozoites; Macro, macrogamonts; Micro, microgamonts. (J to L) Gene expression of a predicted transporter protein (cgd2_800) (J), the microgametocyte-specific gene HAP2 (cgd8_2220) (K), and a macrogamont oocyst wall protein 8 (cgd6_200) (L) at specified times postinfection with C. parvum sporozoites. Gene expression profiles are from a single experiment with three replicates per time point. Values are plotted as the means ± SD.

There was significant recovery in parasite growth for all compounds at their respective EC_50_s after washout versus continuous treatment (Fig. 2C to G). Since the EC_50_, by definition, would be expected to inhibit only 50% of growth after 48 h of treatment, it was not surprising to see recovery in parasite numbers after washout. However, some compounds were more reversible than others at their respective EC_50_; KDU691 only showed an ∼10% recovery in parasite numbers in washout transwells versus continuous treatment (Fig. 1C), whereas MMV665917 and BRD8494 both demonstrated an ∼30% recovery in growth after washout (Fig. 1D and G). Interestingly, parasite growth was irreversible following washout for KDU691, MMV665917, and BRD7929 at their respective EC_90_s (Fig. 1C to E), while AN7973 and BRD8494 showed significant recovery after washout at the EC_90_ (Fig. 1F and G). However, no compound exhibited recovery after washout at its respective 3× EC_90_ value (Fig. 1C to G), indicating that all compounds are parasiticidal at higher concentrations. Most compounds were not toxic to ALI cultures even at 3× the EC_90_, with the exception of MMV665917 that showed reduced host cell viability at this concentration (see Fig. S1 in the supplemental material).

10.1128/mBio.00052-20.1 FIG S1 (A to E) Host toxicity following treatment with KDU691 (A), MMV665917 (B), BRD7929 (C), AN7973 (D), or BRD8494 (E). Host genome equivalents were quantified by qPCR analysis on DNA harvested from transwells at 5 days postinfection. Treatment was conducted with the EC_50_, EC_90_, and 3× the EC_90_ for 48 h, followed by the replacement of medium without compound for an additional 72 h, referred to as washout (W), or following continuous treatment (C). Values represent means ± SD for four technical replicates from two independent experiments. All samples were compared to the control (Cntl), and statistical analysis was performed using one-way ANOVA, followed by a Dunnett’s multiple-comparison test (** *P < *0.01, all other comparisons nonsignificant). Download FIG S1, TIF file, 1.2 MB. Copyright © 2020 Funkhouser-Jones et al. 2020 Funkhouser-Jones et al. This content is distributed under the terms of the Creative Commons Attribution 4.0 International license.

### Delineating life cycle progression of C. parvum in HCT-8 cells.

The differences in reversibility at defined potency suggests that the compounds have different mechanisms of inhibiting C. parvum growth. To determine if they target different life cycle stages of the parasite, we developed microscopy-based assays to examine the timing of inhibition (Fig. 2A). In both HCT-8 and ALI cultures, C. parvum undergoes asexual development and formation of gamonts; however, fertilization is blocked in HCT-8 cultures (20), while it proceeds to oocyst development in ALI cultures (22). To determine the stage against which each compound is most active, we developed methods to define the proportion of each stage present at defined time points during infection. We performed these experiments in HCT-8 cells since the infection is easier to synchronize and visualize by immunofluorescence (IF) microscopy. Infections were synchronized by infection with excysted sporozoites for 2 h and then washing the cultures twice to remove extracellular parasites. Starting 6 hours postinfection (hpi), the thymidine analog EdU was added to cultures in 2-h intervals over a 48-h infection window. Following the EdU pulse, cells were fixed and labeled with an anti-C. parvum antibody (referred to as anti-Cp or Cp), and EdU incorporation into parasite DNA was visualized using click chemistry. Parasites were manually counted for each time point and binned into life stages based on the following criteria: those with a single nucleus were classified as trophozoites (Fig. 2B); type I meronts had either 2 nuclei (Fig. 2C), 4 EdU^+^ nuclei (Fig. 2D), or 8 nuclei (Fig. 2E); type II meronts had four EdU*^−^* nuclei (Fig. 2F); microgamonts contained more than 8 nuclei (Fig. 2G); and macrogamonts lacked a well-defined nucleus and contained oocyst wall-forming bodies visible by phase contrast microscopy (Fig. 2H).

Trophozoites were exclusively present in the cultures up until 8 hpi; they transitioned to type I meronts by 12 hpi before egressing at 16 hpi and reinvading to commence a second round of asexual merogony that was completed by 24 hpi (Fig. 2I). Type II meronts started appearing as early as 28 hpi and only represented a small fraction of parasites in the culture. However, there are presently no good markers to distinguish between early type I and immature type II meronts, both of which would have four EdU^+^ nuclei. Thus, it is possible that our assay underestimates the true number of type II meronts in the culture. Microgamonts appeared around 36 hpi, and macrogamonts with distinguishable wall-forming bodies were visible at ∼40 hpi and outnumbered microgamonts by 44 hpi. C. parvum gene expression markers confirmed the timing of specific stages in the culture. For example, expression of a transporter gene (cgd2_800) was upregulated at 12 and 20 hpi when type I meronts were most prevalent in the culture but declined over time as gamont differentiation predominated (Fig. 2J). Conversely, the Hap2 gene (cgd8_2220), a microgamont-specific gene that is conserved in male gametes of many eukaryotic species, including *Plasmodium* spp. (28, 29), turned on at 36 hpi when microgamonts started to form (Fig. 2K). Similarly, macrogamont-specific genes such as oocyst wall protein 8 (cgd6_200) were highly expressed 48 hpi when macrogamonts dominated the culture (Fig. 2L).

### Determining the effective treatment window for anti-C. parvum compounds.

**FIG 3:**
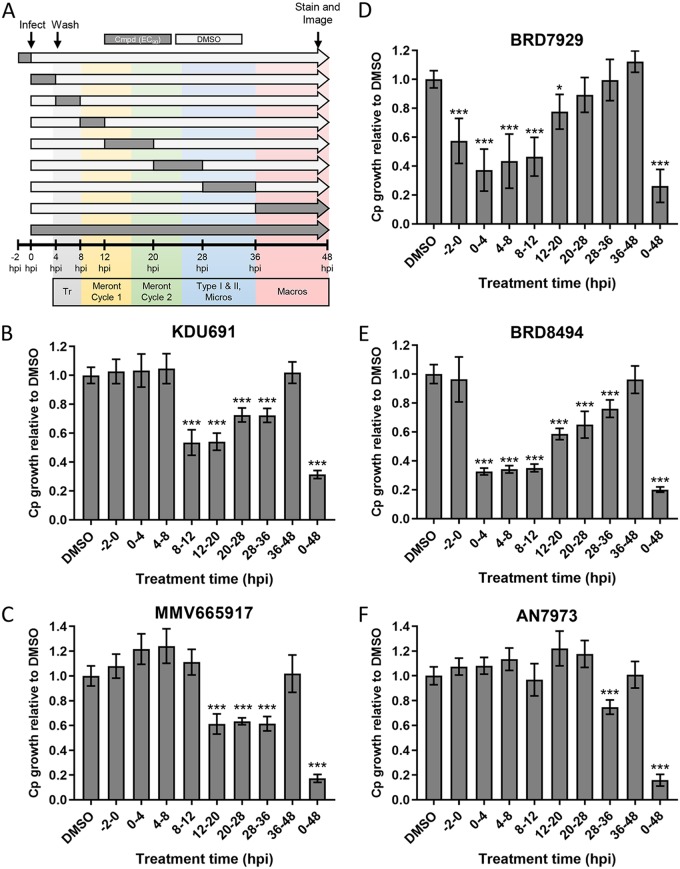
Sliding window analysis of compound effects on different stages of the C. parvum life cycle in HCT-8 cells. (A) Diagram of experimental design in which compounds were added at their respective HCT-8 EC_90_s at specified time intervals (dark gray bars) preinfection (−2 h to 0 h) or postinfection with C. parvum oocysts. All wells were washed 4 hpi to remove unexcysted oocysts. After each treatment window, wells were washed then cultured in medium without compound (cmpd) (light-gray bars) for the remainder of the experiment. At 48 hpi, all wells were fixed and labeled with anti-Cp, followed by goat anti-rabbit Alexa Fluor 488. The number of C. parvum cells in each well was imaged and counted on a Cytation 3 imager and normalized to DMSO-treated control wells. Approximate timing of C. parvum developmental stages is indicated by colored bars. Tr, trophozoites; Micros, microgamonts, Macros, macrogamonts. (B to F) Ratio of Cp growth relative to DMSO controls for AN7973 (B), KDU691 (C), MMV665917 (D), BRD7929 (E), and BRD8494 (F) at their respective EC_90_s during the indicated time windows postinfection. Each bar represents the mean ± SD for six replicates in total from two independent experiments. Data were analyzed with a one-way ANOVA, followed by Dunnett’s multiple-comparison test (*, *P < *0.05; ***, *P < *0.001).

To examine the stage(s) in the C. parvum life cycle that each compound affected, we performed a sliding window analysis of treatment using the EC_90_ in HCT-8 cells (Table 1 and Fig. 3A). Treatment time windows were chosen to target specific steps in the C. parvum life cycle, as summarized in Fig. 3A. Treatment with KDU691 significantly inhibited C. parvum growth starting 8 hpi, indicating that it may block merozoite replication or development (Fig. 3C). In contrast, MMV665917 inhibited growth after the first round of asexual merogony was complete, indicating that it may affect parasite egress and/or merozoite reinvasion (Fig. 3D). The compounds that showed the broadest spectrum of stage inhibition were BRD7929 (Fig. 3E) and BRD8494 (Fig. 3F). Both significantly reduced C. parvum growth compared to dimethyl sulfoxide (DMSO)-treated controls at almost every time point, indicating that they likely block multiple biological processes or one process shared by multiple stages of parasite development. AN7973 was not active during most of the individual treatment windows and only had a slight, but significant, effect on parasite growth at 28 to 36 hpi (∼25% inhibition). In contrast, continuous culture with the compound for the full 48 h inhibited parasite growth by ∼85% (Fig. 3B). This indicates that C. parvum needs to be exposed to AN7973 for longer than the 4- to 12-h treatment windows in order to be effective. Similarly, treatment with control compound nitazoxanide at the EC_90_ had little effect on parasite growth for most short time points (Fig. S2A); however, such treatment for the full 48 h was completely cytotoxic to the host cells (Fig. S2B). Previous studies have also emphasized the potential toxicity of nitazoxanide, limiting its clinical usefulness (30). None of the other compounds exhibited host toxicity at the EC_90_ over the same time window (Fig. S2B).

10.1128/mBio.00052-20.2 FIG S2 (A and B) Effect of nitazoxanide treatment at EC_90_ (6.5 μM) in infected HCT-8 cells on C. parvum growth in a sliding window analysis (see Fig. 3A for experimental setup) (A) and host toxicity compared to other compounds at their EC_90_ values after 48 h of treatment (B). Each bar represents the mean ± SD for six replicates total from two independent experiments. Data were analyzed with a one-way ANOVA, followed by Dunnett’s multiple-comparison test comparing each experimental mean to the DMSO control (**, *P < *0.01; ***, *P < *0.001). Download FIG S2, TIF file, 0.3 MB. Copyright © 2020 Funkhouser-Jones et al. 2020 Funkhouser-Jones et al. This content is distributed under the terms of the Creative Commons Attribution 4.0 International license.

### EdU pulsing to further dissect the stage specificity of anti-*Cryptosporidium* compounds.

**FIG 4:**
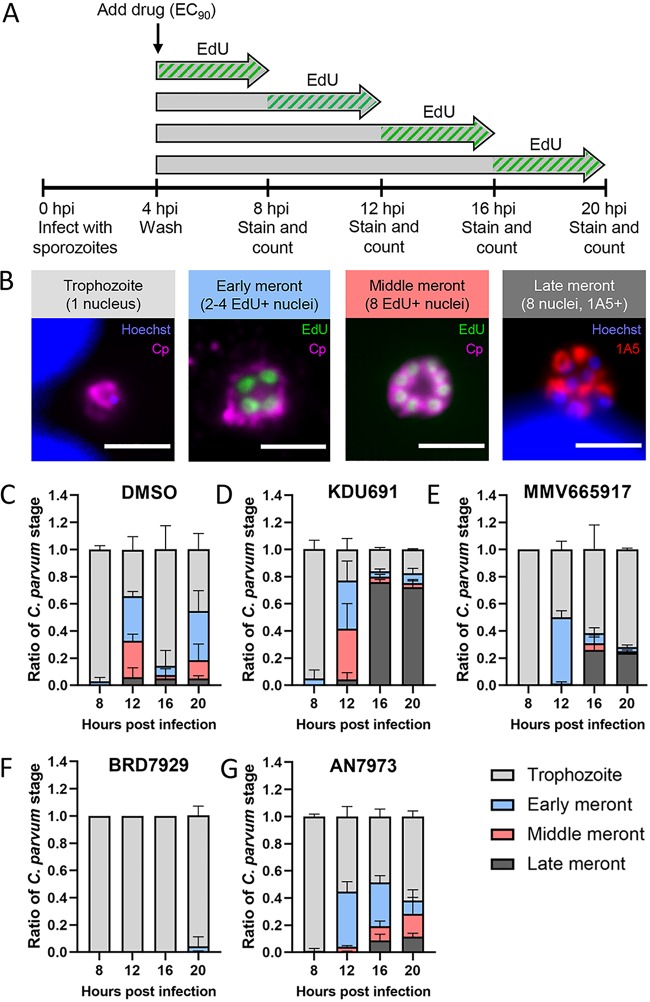
Use of EdU labeling to define effects of compounds on different stages of C. parvum asexual replication. (A) HCT-8 cells plated on coverslips were infected with excysted sporozoites for 4 h before compound was added at the EC_90_. EdU was added to culture medium starting at 4, 8, 12, or 16 hpi. Coverslips were fixed and stained after 4 h of incubation with EdU. (B) Immunofluorescence assay (IFA) images defining the progression of asexual replication using EdU- and stage-specific antibodies. Single-nucleus trophozoites undergo two rounds of DNA replication to form “early meronts” with 2 or 4 EdU^+^ nuclei (green). A third round of DNA replication generates “middle meronts” with 8 EdU^+^ nuclei, which then mature into “late meronts” containing 8 individual merozoites that each label with monoclonal antibody 1A5 (red) in a polarized manner. Scale bars = 3 μm. (C to G) Quantification of the proportion of each asexual stage present at the indicated time points as defined with EdU and 1A5 labeling for the DMSO control (C), KDU691 (D), MMV665917 (E), BRD7929 (F), and AN7973 (G). Error bars represent the mean ± SD for three biological replicates for each compound and 6 biological replicates for the DMSO control.

We combined EdU pulsing with a monoclonal antibody that labels mature merozoites, 1A5 (24), to better understand the specific biological processes inhibited by each compound. Infected HCT-8 cultures were treated with compounds at the EC_90_, and EdU was added to individual cultures in 4-h increments until 20 hpi (Fig. 4A). At the end of each 4-h EdU pulse, coverslips were fixed and labeled with 1A5, anti-C. parvum (anti-Cp), and click chemistry to label EdU incorporation into newly synthesized DNA. Life cycle stages were then defined based on the number and EdU status of their nuclei and whether the 1A5 marker was present, as follows: trophozoites had one nucleus, early stage meronts had either two or four EdU^+^ nuclei, middle meronts had eight EdU^+^ nuclei but no 1A5 labeling, and late meronts had eight nuclei and positive 1A5 labeling indicative of merozoite development (Fig. 4B and Fig. S3).

10.1128/mBio.00052-20.3 FIG S3 EdU pulse assays to define effects of compounds on different stages of C. parvum asexual replication. Images shown are individual channels for the merged images that are presented in Fig. 4B. DNA was stained with Hoechst (blue), replicating DNA was visualized with EdU (green), mature meronts are recognized by monoclonal antibody 1A5 (red), and all C. parvum cells were stained with polyclonal Pan Cp (magenta). Scale bars = 3 μm. Download FIG S3, TIF file, 0.8 MB. Copyright © 2020 Funkhouser-Jones et al. 2020 Funkhouser-Jones et al. This content is distributed under the terms of the Creative Commons Attribution 4.0 International license.

As expected, DMSO control cultures showed a cyclical pattern of DNA replication and merozoite reinvasion, with trophozoites dominating the cultures at 8 hpi and 16 hpi, followed by meronts at 12 hpi and 20 hpi (Fig. 4C). In contrast, KDU691-treated cultures stalled at the late meront stage at 16 hpi, indicating a possible block in merozoite maturation or egress (Fig. 4D). MMV665917-treated cultures lagged in development compared to the DMSO control and still contained a majority of trophozoites at 20 hpi (Fig. 4E). Treatment with BRD7929 completely blocked DNA replication (nearly all parasites contained a single EdU-negative nucleus) up until 20 hpi (Fig. 4F). Last, AN7973-treated cultures showed a lag in development, with fewer parasites continuing to the late meront stage (Fig. 4G). These results were intriguing considering that a recent study reported that AN7973 completely blocked DNA synthesis as detected by a lack of EdU incorporation in compound-treated C. parvum (15). This prior study used a higher effective concentration of compound (2× the EC_90_) and only evaluated a single time point (11 hpi). When we repeated the EdU pulse experiment for AN7973 at both the EC_90_ and 2× the EC_90_, we found that DNA replication was blocked at the higher concentration until 16 hpi, when ∼15% of the parasites had two or more EdU^+^ nuclei (Fig. S4). Our results corroborate the previous study since we did not detect DNA replication at 12 hpi when treated at 2× the EC_90_; however, our findings also reveal that some parasites recover at the higher dose given additional time despite constant drug pressure.

10.1128/mBio.00052-20.4 FIG S4 Inhibition of parasite replication by AN7973. (A) Quantification of the proportion of each asexual stage present at the indicated time points as defined with EdU and 1A5 staining. Error bars represent mean ± SD (*n* = 2 biological replicates). (B) IFA images of DMSO or parasites treated with AN7973 at the EC_90_ or 2× the EC_90_. HCT-8 cultures were infected with ∼8 × 10^6^ excysted sporozoites, treated during the entire culture period, fixed, and stained at 12 or 20 hpi with EdU (green), polyclonal Pan Cp (red), and Hoechst DNA stain (blue). Scale bars = 5 μm. Download FIG S4, TIF file, 1.7 MB. Copyright © 2020 Funkhouser-Jones et al. 2020 Funkhouser-Jones et al. This content is distributed under the terms of the Creative Commons Attribution 4.0 International license.

### KDU691 treatment impedes individual merozoite formation.

**FIG 5:**
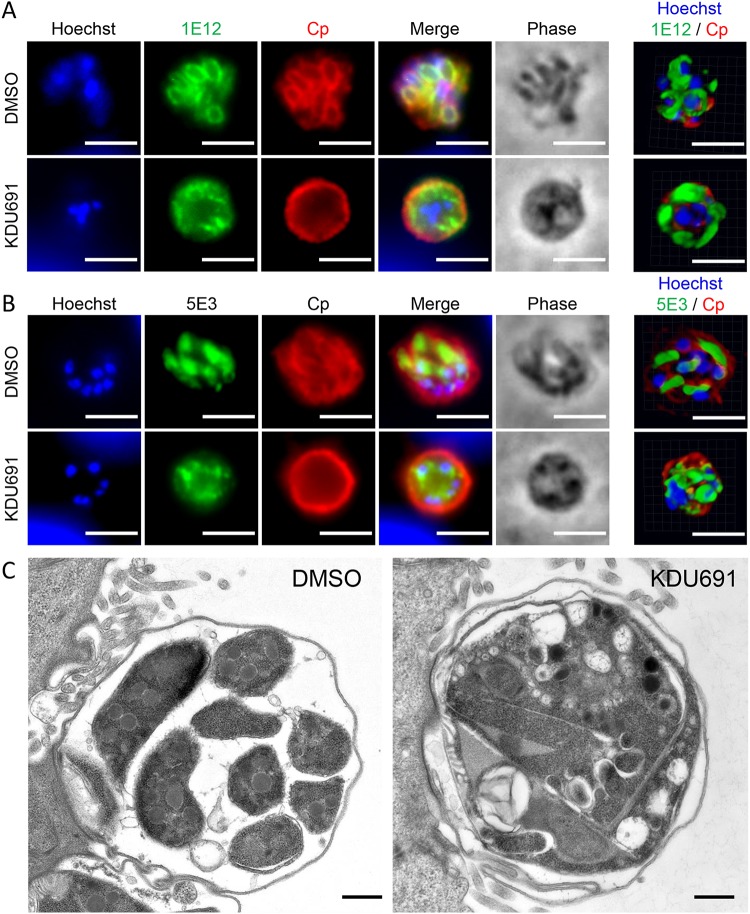
Influence of KDU691 on merozoite maturation. (A and B) IFA images of DMSO- or KDU691-treated parasites from HCT-8 cultures infected with excysted sporozoites fixed and labeled at 22 hpi with anti-Cp (red), Hoechst DNA stain (blue), and either monoclonal antibody 1E12 (green), which localizes to the parasite membrane (A) or monoclonal antibody 5E3, which recognizes the apical end of individual merozoites (B). Images on the right are 3D renderings from confocal z-stacks of parasites labeled with the same antibodies but from independent experiments from the images on the left. Scale bars = 3 μm. (C) Transmission electron micrographs of DMSO- or KDU691-treated parasites at 22 hpi. Scale bars = 500 nm.

To further define the block in growth in KDU691-treated cultures, we used immunofluorescence (IF) imaging and transmission electron microscopy (TEM) to examine merozoite development. Monoclonal antibody 1E12, which localizes to C. parvum membranes (24), labeled the membranes of individual merozoites in DMSO control cultures at 22 hpi but remained localized around the perimeter of meronts in KDU691-treated cultures (Fig. 5A). Furthermore, monoclonal antibody 5E3, which recognizes the apical pole of excysted sporozoites and individual merozoites in late meronts (24), was detectable at the poles of merozoites in DMSO cultures but showed more diffuse labeling throughout the cytosol in KDU691-treated cultures (Fig. 5B). When examined by electron microscopy, KDU691-treated meronts contained aberrant membrane invaginations and lacked separate merozoites compared to DMSO control parasites (Fig. 5C). Taken together, these results indicate that KDU691 inhibits merozoite formation, perhaps by impeding the separation of membranes surrounding individual merozoites after DNA replication has occurred. Combined with the accumulation of late-stage meronts as judged by nuclear staining (Fig. 4D), these results indicate that nuclear division is not affected but that egress is likely blocked by the failure of individual merozoites to form in KDU691-treated cultures.

### MMV665917 inhibits macrogamont development.

**FIG 6:**
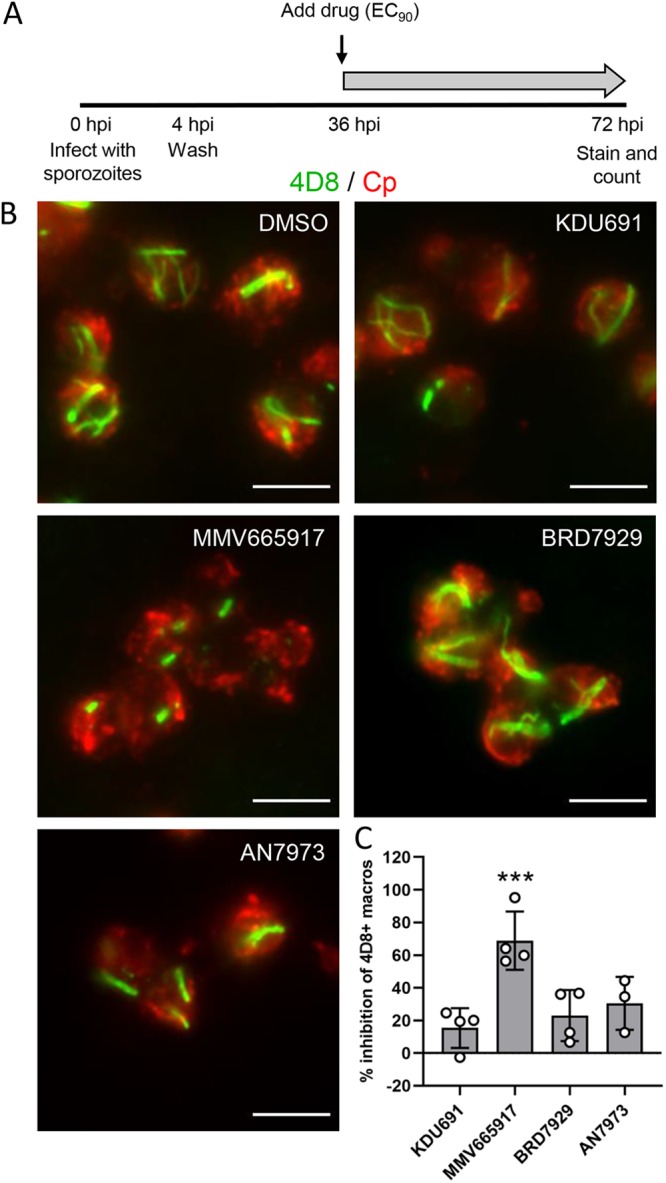
Effect of compounds on formation of macrogamonts. (A) HCT-8 cells plated on coverslips were infected with excysted sporozoites and then washed at 4 hpi to remove extracellular parasites. Compounds were added at their respective EC_90_s starting at 36 hpi, and cells were fixed and labeled at 72 hpi. (B) IFA images of macrogamonts treated with the indicated compound and labeled with monoclonal antibody 4D8 (green) and anti-Cp (red). Scale bars = 5 μm. (C) Percent inhibition of 4D8^+^ macrogamonts present in cultures treated with the indicated compound compared to DMSO control cultures. Error bars represent the mean ± SD of the results from three independent experiments. Data were analyzed using a nonparametric Kruskal-Wallis test, followed by Dunn’s multiple-comparison test that compared each compound to the DMSO control (***, *P < *0.001).

Although MMV665917 delayed meront development in the EdU pulse assay (Fig. 4E), there were no obvious defects in merozoite formation based on labeling with 1E12 (Fig. S5A) or 5E3 (Fig. S5B). A previous study found that MMV665917 acted predominantly against the sexual stages of C. parvum based on antibody labeling of a meiosis-specific protein, DMC1 (17). To examine macrogamont formation in our cultures, we utilized monoclonal antibody 4D8, which recognizes elongated fibrillar structures in macrogamonts (24). Infected cultures were cultured without compound for 36 hpi to allow for normal asexual development before adding compounds at their respective EC_90_s for an additional 36 h of culture (Fig. 6A). The labeling pattern of 4D8 was used to quantify the percent inhibition of macrogamont formation after compound treatment. Macrogamonts were considered 4D8^+^ if they showed any striated structure that labeled with 4D8, regardless of length or branching pattern. Macrogamonts treated with KDU691, BRD7929, or AN7973 had 4D8 labeling patterns in both length and branching similar to those in the DMSO control, while 4D8^+^ structures in MMV665917 macrogamonts were much shorter and unbranched (Fig. 6B). When the ratio of macrogamonts to total C. parvum was quantified for each compound and expressed as a percentage of inhibition compared to the DMSO control, MMV665917 was the only compound that significantly inhibited the development of 4D8^+^ macrogamonts (Fig. 6C).

10.1128/mBio.00052-20.5 FIG S5 Analysis of sexual-stage division during treatment with MMV665917. (A and B) IFA images of DMSO- or MMV665917-treated parasites. HCT-8 cultures were infected with ∼2 × 10^6^ excysted sporozoites, treated for the remaining duration of culture at the EC_90_ (9.54 μM), fixed, and stained 22 hpi with Pan Cp (red), Hoechst DNA stain (blue) and either monoclonal antibody 1E12 (green), which localizes to the parasite membrane (A), or monoclonal antibody 5E3, which recognizes the apical end of individual merozoites (B). Scale bars = 3 μm. Download FIG S5, TIF file, 1.3 MB. Copyright © 2020 Funkhouser-Jones et al. 2020 Funkhouser-Jones et al. This content is distributed under the terms of the Creative Commons Attribution 4.0 International license.

### BRD7929 treatment blocks nuclear replication and increases feeder organelle area.

**FIG 7:**
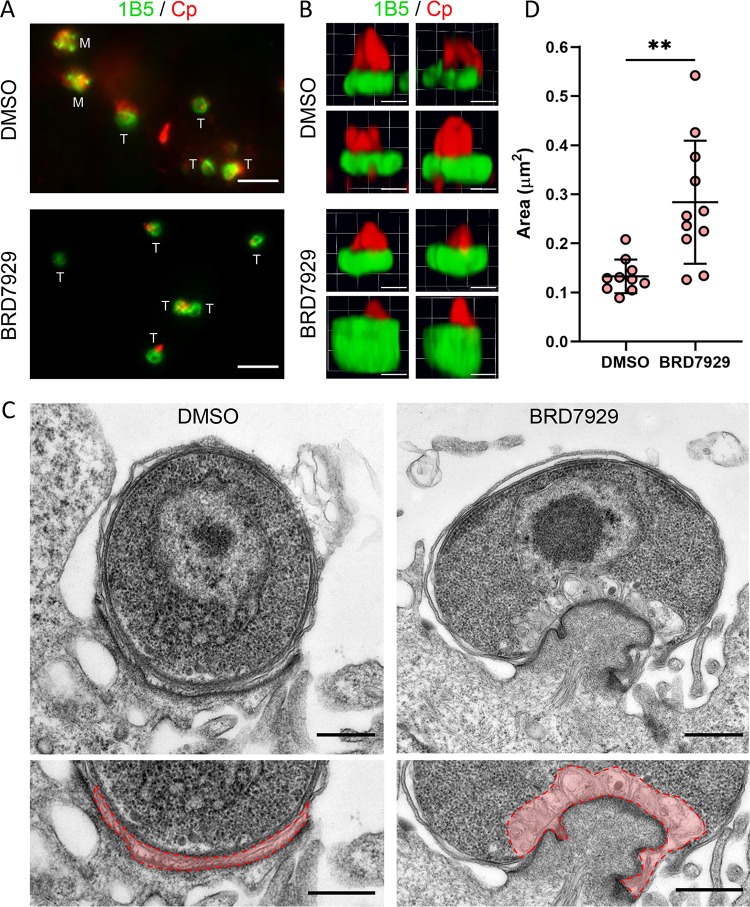
Effect of BRD7929 on parasite replication and formation of the feeder organelle. (A) IFA images of DMSO- or BRD7929-treated parasites from HCT-8 cultures infected with excysted sporozoites fixed and labeled at 12 hpi with anti-Cp (red) and monoclonal antibody 1B5 (green), which localizes to the host-parasite interface. The stages of individual parasites are indicated with “M” for type I meronts or “T” for trophozoites. Scale bars = 5 μm. (B) Three-dimensional renderings from confocal z-stacks of DMSO- or BRD7929-treated parasites from HCT-8 cultures infected with excysted sporozoites fixed and labeled at 8 hpi with 1B5 and rabbit polyclonal anti-Cp (red). Images represent four parasites per treatment group from the same experiment. Scale bars = 1 μm. (C) Transmission electron micrographs of DMSO- or BRD7929-treated parasites at 12 hpi. Bottom images are enlarged sections of the top images with the feeder organelles outlined and false-colored red. Scale bars = 500 nm. (D) Area in μm^2^ of the feeder organelles of trophozoites from images from the same experiment as panel C. *n* = 10 trophozoites for DMSO and *n* = 11 trophozoites for BRD7929. Data were analyzed using an unpaired, two-tailed Student *t* test (**, *P < *0.01).

BRD7929-treated parasites showed a nearly complete block of EdU incorporation (Fig. 4F), suggesting that this compound blocks DNA replication. To test this prediction, we labeled with monoclonal antibody 1B5, which recognizes the base of trophozoites in a unique doughnut pattern (24), at 12 hpi when the type I meronts should be maturing (Fig. 3A). Consistent with this prediction, many parasites in the DMSO control cultures had progressed to the mature type I meront stage, whereas only trophozoites were present in BRD7929-treated cultures (Fig. 7A). Interestingly, we observed expanded 1B5 labeling at the base of BRD7929-treated trophozoites compared to DMSO control cultures when viewed as three-dimensional (3D) renderings of transverse confocal z-stacks through the parasites (Fig. 7B). Since 1B5 likely recognizes an antigen at the host-parasite interface (24), we analyzed infected DMSO- or BRD7929-treated cultures at 12 hpi to determine whether we could observe morphological differences by transmission electron microscopy. To capture parasites with similar orientations from the two treatment groups, we specifically imaged single-nucleus trophozoites sitting on electron-dense pedestals with a sizable host-parasite interface (Fig. 7C). BRD7929-treated trophozoites exhibited normal membrane and nuclear architecture comparable to those from DMSO control cultures (Fig. 7C). However, compound-treated parasites had expanded feeder organelles (Fig. 7C, highlighted in pink) that were significantly larger in area than those from DMSO trophozoites (Fig. 7D). These findings are consistent with BRD7929 blocking parasite replication and stalling growth at the early trophozoite stage.

## DISCUSSION

Although several new inhibitors of *Cryptosporidium* sp. growth have been identified in high-throughput screening efforts, we lack good methods for defining their mechanisms of action. To begin alleviating this problem, we have developed several *in vitro* assays to profile the development of life cycle stages that occur *in vitro* and to define static versus cidal activities. Taking advantage of air-liquid interface cultures that allow complete parasite development, we define concentration- and time-dependent treatment conditions that provide irreversible inhibition. Using a combination of EdU labeling of replicating nuclei and monoclonal antibodies to specific stages, we define the progression of life cycle development that occurs in HCT-8 cells. By combining pulsed compound treatment for defined intervals with EdU pulse labeling, we were able to identify key points in the life cycle that were affected by distinct classes of inhibitors. Among the four classes of compounds studied here were those that block nuclear division (PheRS inhibitors), prevent merozoite formation and hence block egress (PI4K inhibitors), or block macrogamont development (a piperazine inhibitor of unknown function). Collectively, these assays provide a set of guidelines for evaluating future compounds to define the stages of the life cycle that they affect and for defining time- and concentration-dependent killing.

One of the limitations of using transformed cell lines such as HCT-8 cells for growing C. parvum is that the parasites do not proceed past gamont development (20), and parasite numbers decline over time after reaching a peak between 48 and 72 hpi (31, 32). Although previous studies have tried to estimate “time-to-kill” rates in response to treatment in HCT-8 cells (8, 17), it is not possible to perform classical washout and recovery experiments using such transformed lines due to the fact that replication ceases after a few days. In contrast, C. parvum undergoes complete development, including the production of viable oocysts in ALI cultures (22). Moreover, infected ALI cultures can be propagated for up to 3 weeks (22), providing an experimental system for treatment and recovery experiments. We utilized ALI cultures of C. parvum to examine the four classes of compounds studied here using increasing concentrations from the EC_50_, to the EC_90_, and to 3× the EC_90_ for a duration of 48 h. All compounds showed partial recovery following treatment at the EC_50_, a result expected from the fact that this concentration should block growth only by 50%. In contrast, raising the concentration to the EC_90_ resulted in a complete absence of recovery for KDU691, MMV665917, and BRD7929. Somewhat surprisingly, treatment with BRD8494 resulted in partial recovery at the EC_90_, despite the fact that this compound is very closely related to BRD7929 and likely shares the same PheRS target (16), although this has not been formally demonstrated in *Cryptosporidium* spp. The difference in potency may be a result of the greater hydrophobicity of BRD7929, such that removal by the replacement of medium during the washout phase may be less effective. Additionally, the benzoxaborole AN7973 showed partial recovery even when treated at the EC_90_ and 2× the EC_90_. This compound was also much less effective than either PheRS or PI4K inhibitors when used for short treatment windows. The longer time requirement for inhibition by AN7973 is consistent with a recent report that estimated the half-life (*t*_1/2_) for killing to be on the order of 9 h (15). Although we have not extended our studies to *in vivo* treatment, the concentration- and time-dependent findings may be useful in estimating exposure levels that would be needed to achieve complete killing *in vivo*.

A recent study developed a suite of assays for profiling the inhibition of C. parvum growth using a combination of cellular assays (17). Here, we extend these assays to examine additional details of the life cycle based on shorter EdU pulses to label replicating nuclei combined with monoclonal antibodies to stage-specific antigens (24). Several of our findings help extend the toolkit for analyzing the life cycle and for deconvolving cellular pathways targeted by different inhibitors. First, the use of short EdU pulses indicates that, in HCT-8 cells, C. parvum growth proceeds through two rounds of type I merogony that precedes type II merogony and the appearance of macrogamonts and microgamonts. Second, using antibodies to apical antigens that appear late in merogony (24), we were able to distinguish immature from mature type I meronts as well as type II meronts. It is generally believed that type II meronts give rise to gamonts (33), which makes the low abundance of type II meronts in our cultures surprising given the significant number of gamonts that appear after 36 hpi. However, it is possible that our methods underestimate the true frequency of type II meronts, as there are no known stage-specific markers that are exclusive to type II meronts. Further studies to identify such markers may help resolve this apparent discrepancy. Finally, by using short treatment pulses that were designed to pinpoint distinct life cycle stages, we were able to separate the inhibitor classes in terms of when they are most active.

The compounds BRD7929 and BRD8494, which likely target PheRS, were the most potent in terms of acting rapidly across multiple stages, including preventing nuclear division and thus blocking type I merogony development. In contrast, KDU691, which likely targets PI4K, and MMV665917, which acts on an unknown target, had almost no effect on replicating type I meronts and were only effective when added later in the cycle at the boundary of type I/type II merogony or as sexual stages emerge. By further dissecting the development of type I merogony, it was evident that KDU691 resulted in accumulation of a late-stage type I meront, while the BRD7929 completely blocked nuclear division, arresting parasites at the early trophozoite stage. Finally, both AN7973 and MMV665917 delay the progression of later stages but do not block DNA replication.

By extending our analysis to use monoclonal antibodies that recognize specific structures in the parasite that help define the life cycle, combined with transmission electron microscopy to examine the cellular ultrastructure, we were able to confirm the findings described above and provide a cellular context for understanding the different mechanisms of individual compounds. For example, labeling with antibodies that recognize the apical pole in mature merozoites revealed that KDU691 prevents the separation of merozoites into individual daughter cells. A similar finding has been described previously for P. falciparum, where treatment with a related imidazopyrazine led to multinucleate schizonts that failed to segment into merozoites (12). Consequently, it is likely that KDU691 prevents the egress of merozoites from type I meronts, thus stalling the cycle at this asexual replicating phase. In contrast, MMV665917 has no effect on merozoite division, and fully functional type I meronts are formed, although they are slightly delayed in development. However, the major effect of MMV665917 appears to be aberrant macrogamont formation, as revealed by labeling with monoclonal antibody (MAb) 4D8 that detects a striated fiber that forms in macrogamonts (24). Our finding corroborates a previous report that reached a similar conclusion using a different antibody to macrogamonts (17). However, pulsed treatments with MMV665917 at earlier time points also decreased the number of asexual parasites, indicating that this compound acts on both asexual and sexual development in C. parvum. As the target of MMV665917 is unknown, further work is needed to determine whether it targets multiple pathways that independently inhibit asexual and sexual stages or a single pathway shared between the two stages. Finally, the most potent compound that we examined was BRD7929, which completely blocked parasite replication, stalling the parasite at the trophozoite stage. This bicyclic azetidine likely targets PheRS based on studies conducted in P. falciparum (16), suggesting that it prevents the synthesis of proteins critical for DNA replication. Treated parasites remain intact for 20 hpi, and the only morphological defect that they displayed was an enlarged feeder organelle, a highly membranous region at the host-parasite interface that is thought to be responsible for the transport of nutrients (34, 35). The enlargement of the feeder organelle suggests that some functions may persist in treated parasites (i.e., transport) despite the block in replication. Nonetheless, treatment with BRD7929 was effective when given for short time intervals, and, when treated for 48 h at the EC_90_, parasites did not recover from treatment, indicating that the compound is cidal under these conditions.

Cryptosporidiosis has recently been recognized as an important cause of diarrheal disease in infants in the developing world (3, 36). Treatment is limited by the fact that the only existing FDA-approved drug, nitazoxanide, is not approved for infants, is not effective in immunocompromised patients (37), and has varied efficacy in immunocompetent individuals (38). As such, there has been a concerted effort to identify new compounds that are potent and selective inhibitors of *Cryptosporidium* sp. growth as potential leads for the development of new drugs (39). To contribute toward this goal, we have developed methods that can be used to define cidal activity. Importantly, this outcome varies with the concentration and time of treatment that is necessary to prevent the recovery of parasite growth, parameters that are more easily monitored in long-term ALI cultures that support continuous parasite growth. We also expand the repertoire of probes for determining the life cycle stages where compounds are most effective, revealing several novel modes of action among existing lead compounds. These tools should aid future studies evaluating differences in potency, selectivity, mechanism of action, and potential synergy when establishing metrics to achieve *in vivo* efficacy of new drugs for the treatment of cryptosporidiosis.

## MATERIALS AND METHODS

### Preparation of oocysts.

C. parvum oocysts were obtained from the Witola lab (University of Illinois at Urbana-Champaign). The C. parvum isolate (AUCP-1) was maintained by repeated passage in male Holstein calves and purified from fecal material, as described previously (40). Animal procedures were approved by the Institutional Animal Studies Committee at the University of Illinois at Urbana-Champaign.

Purified oocysts were stored at 4°C in phosphate-buffered saline (PBS) plus 50 mM Tris and 10 mM EDTA (pH 7.2) for up to 6 months. Before infection, C. parvum oocysts were treated in a 40% bleach solution (commercial bleach containing 8.25% sodium hypochlorite) diluted in Dulbecco’s phosphate-buffered saline (DPBS; Corning Cellgro) for 10 min on ice. Oocysts were then washed three times in DPBS containing 1% (wt/vol) bovine serum albumin (BSA; Sigma) before storing at 4°C in DPBS containing 1% BSA for up to 2 weeks before infection. For some experiments, oocysts were excysted in 0.75% sodium taurocholate at 37°C for 1 h. The excysted sporozoites were centrifuged at 2,500 rpm for 3 min and then resuspended in culture medium prior to use.

### Compounds.

Previously characterized inhibitors of C. parvum growth were obtained from the following organizations: compound MMV665917 was obtained from the University of Vermont, compound AN7973 was obtained from Calibr, compound KDU691 was obtained from Novartis, and compounds BRD7929 (full name, BRD-K78727929-001-03-2) and BRD8494 (full name, BRD-K21118494-001-01-1) were obtained from the Broad Institute. Compounds were dissolved at 10 mM in DMSO and stored at –80°C until use. For use in biological assays, compounds were diluted in culture medium to a final concentration of 1% DMSO and compared to medium containing only 1% DMSO as a control.

### HCT-8 cell culture.

Human ileocecal adenocarcinoma cells (HCT-8 cells; ATCC CCL-244) were maintained in RPMI 1640 medium (Gibco, ATCC modification) supplemented with 10% fetal bovine serum. Cells were confirmed to be mycoplasma free with the e-Myco plus *Mycoplasma* PCR detection kit (Boca Scientific).

### EC_50_ determination in HCT-8 cells.

HCT-8 cells plated on 96-well optically clear plates (Greiner Bio-one 655090; Fisher) were infected with oocysts the day after cells reached confluence. Compounds were added in culture medium containing 1% DMSO to generate a 9-point dilution curve to determine their EC_50_ values. Twenty-four hours after compound addition, samples were fixed in 4% formaldehyde for 10 min, washed twice with PBS, permeabilized, and blocked in 0.1% Triton X-100 and 1% BSA in PBS. Samples were labeled with rabbit anti-Cp antibody developed against C. parvum oocysts and sporozoites (22), followed by goat anti-rabbit Alexa Fluor 488 (A11034; Invitrogen) in blocking buffer and incubated for 1 h at room temperature, washed three times with buffer, and stained with 1 μg/ml Hoechst for 10 min. Plates were imaged using a BioTek Cytation 3 cell imager to quantify parasite growth (Alexa Fluor 488 label) and monitor host cell viability (Hoechst nuclear staining). EC_50_ and EC_90_ values were calculated using a nonlinear regression curve fit (log inhibitor versus normalized response – variable slope) with three technical replicates per experiment using the Prism 8 software (GraphPad). The mean EC_50_ and EC_90_ per compound are expressed as average values from three independent experiments.

### Air-liquid interface culture.

The conditions for generating the ALI monolayer system have been defined in greater detail previously (23). Irradiated 3T3 mouse fibroblast (i3T3) cells were plated on transwells (polyester membrane, 0.4-μm pore; Corning Costar, with 12 transwells per 24-well plate) coated with 10% Matrigel (Corning) at a density of 8 × 10^4^ i3T3 cells per transwell. DMEM-high-glucose medium supplemented with 10% fetal bovine serum, 100 U/ml penicillin, and 0.1 mg/ml streptomycin was added to the top (200 μl) and bottom (400 μl) of the transwells and incubated at 37°C for 24 h. Mouse intestinal epithelial cell (mIEC) spheroids were trypsinized and plated on the i3T3 feeder layer the next day (5 × 10^4^ mIECs per transwell). The medium was then changed to 50% L-WRN conditioned medium (CM) supplemented with 10 μM Y-27632 (ROCK inhibitor), as defined previously (25, 26), at the same volumes mentioned above. The 50% CM plus ROCK inhibitor medium was used for the maintenance of ALI transwells, with transwells receiving fresh top medium (200 μl) and bottom medium (400 μl) every 2 to 3 days. After 7 days, medium from the top compartment was removed to initiate the ALI culture, while the bottom medium was changed every 2 to 3 days to maintain the culture.

### EC_50_ determination and washout experiments in ALI cultures.

ALI monolayers were infected with oocysts on day three after top medium removal and washed at 2 hpi, and a 5-point dilution series of compounds was added to both the top (50 μl) and bottom (400 μl) chambers of the transwell. DNA extraction was performed at 48 h postinfection with the QIAamp DNA minikit (Qiagen). C. parvum growth and host cell viability were tracked by monitoring the expression of their respective glyceraldehyde-3-phosphate dehydrogenase (GAPDH) genes using qPCRs that were run on the QuantStudio 3 real-time PCR system (Thermo Fisher), as described previously (22). EC_50_ and EC_90_ values were calculated using a nonlinear regression curve fit (log inhibitor versus normalized response – variable slope) with two technical replicates per experiment using the Prism 8 software (GraphPad). The mean EC_50_ and EC_90_ values per compound are an average of the values from two independent experiments.

ALI monolayers were also used to monitor recovery after treatment with different concentration of compounds. ALI transwells were infected with oocysts on day three after top medium removal and washed at 2 hpi with DPBS, 50 μl of the compound in 50% CM was added to the top of the monolayer, and 400 μl of the compound in 50% CM was added to the bottom chamber. At 48 hpi, transwells were washed three times with DPBS, and the medium was replaced without compound (50 μl top and 400 μl bottom) for the remaining duration of the experiment. For continuous treatments, medium replacement included fresh compound. DNA extraction was done at 2 dpi and 5 dpi using the QIAamp DNA minikit (Qiagen). C. parvum and host genome qPCRs were analyzed on the QuantStudio 3 real-time PCR system (Thermo Fisher), as described previously (22), using the QuantStudio software. Two replicates from two independent experiments (*n *= 4) were combined for statistical analysis.

### EdU pulsing to define C. parvum life cycle progression in HCT-8 cells.

To reduce the amount of EdU uptake by replicating host cells, HCT-8 cells from confluent cultures were irradiated at 6,000 rad and stored in liquid nitrogen until further use. Thawed irradiated HCT-8 cells were plated on round coverslips in 24-well culture plates and incubated for ∼24 h. HCT-8 monolayers were infected with excysted sporozoites and washed two times with sterile DPBS at 2 hpi to remove extracellular parasites. Starting at 6 hpi, EdU was added to the culture medium at a final concentration of 10 μM for separate 2-h pulses spaced over 48 h before fixing the cells in 4% formaldehyde for 10 min. Coverslips were permeabilized in 0.05% saponin and treated with the Click-iT Plus EdU 488 imaging kit (Thermo Fisher Scientific) to label EdU. Coverslips were then labeled with anti-RH (a polyclonal antibody generated against Toxoplasma gondii that recognizes all intracellular stages of C. parvum [24]), followed by labeling with Alexa Fluor 568 antibody (Thermo Fisher Scientific) and Hoechst staining. To calculate the percentage of parasites in each life stage per time point, the number of parasites at each life stage was counted from 10 fields using a 100× oil immersion objective on a Zeiss Axioskop Mot Plus fluorescence microscope, and then the sum was divided by the total number of C. parvum parasites for that time point.

### Analysis of C. parvum gene expression.

HCT-8 cells were plated in 6-well culture plates and incubated ∼24 h before infection. Monolayers were infected with excysted sporozoites, washed twice with DPBS at 2 hpi, and returned to culture in fresh HCT-8 medium. RNA was collected from three wells per time point in RLT buffer (Qiagen) plus 1% β - mercaptoethanol, homogenized using a QIAshredder column (Qiagen), and then stored at –80°C until further processing. RNA was extracted using the RNeasy minikit (Qiagen), treated with the DNA-free DNA removal kit (Thermo Fisher Scientific), and converted to cDNA using the SuperScript VILO cDNA synthesis kit (Thermo Fisher Scientific). Reverse transcription-quantitative PCR (RT-qPCR) was run on a QuantStudio 3 real-time PCR system (Thermo Fisher Scientific) with TB Green Advantage qPCR premix (TaKaRa Bio) and the following primers (5′ to 3′): Transporter cgd2_800 (forward, TGAAAGCGATACAGATGATGGT; reverse, GTTTGTAGGGATTAGCTGGTCAA) (41), HAP2 cgd8_2220 (forward, TTGGATTCATTAGGAGAAATTGG; reverse, ATGTTGCTACCCAAGACACAGA) (41), Oocyst wall protein 8 cgd6_200 (forward, TGATATGCCCAGAAGGAG; reverse, TTATCTCCTCTCTAGCAACGCA) (41), and C. parvum 18S (forward, TAGAGATTGGAGGTTGTTCCT; reverse, CTCCACCAACTAAGAACGGCC) (42). Relative gene expression was calculated with the ΔΔ*C_T_* method (43) using C. parvum 18S rRNA as the reference gene and normalizing expression to the mean expression of that gene at 4 hpi.

### Sliding window analysis of compound effects on C. parvum in HCT-8 cells.

HCT-8 cells were plated on 96-well optically clear plates (Greiner Bio-one 655090; Fisher) and incubated until confluency (∼24 h). To test the effect of pretreatment, wells were treated with compounds at their EC_90_s in medium containing 1% DMSO for 2 h and then washed three times with DPBS before infection with C. parvum oocysts. The remaining wells were infected with C. parvum oocysts, washed three times with DPBS after 4 h to remove unexcysted oocysts, and returned to culture. Separate wells were treated with compounds at the EC_90_s in medium containing 1% DMSO for defined time intervals after infection (i.e., 4- or 8-h intervals, or continuous treatment). At 48 h postinfection, cells were fixed in 4% formaldehyde for 10 min and then labeled with rabbit anti-Cp, followed by goat anti-rabbit Alexa Fluor 488 and Hoechst dye. The plate was imaged on the BioTek Cytation 3 cell imager to calculate the number of C. parvum parasites (Alexa Fluor 488 signal) and host cells (Hoechst signal) using the Gen 5 software. Experiments represent two biological replicates performed on different days with three technical replicates per biological replicate.

### Determining the stage specificity of compound inhibition using EdU pulsing.

HCT-8 cells were plated on 12-mm-diameter glass coverslips (Thermo Fisher Scientific) in 24-well tissue culture plates and incubated until confluency (∼24 h). Monolayers were infected with excysted sporozoites, washed twice with DPBS at 4 hpi, and treated with compounds at their EC_90_s (Table 1) in 1% DMSO in HCT-8 medium. For the EdU pulse labeling, one set of two coverslips per treatment group was incubated with 10 μM EdU and then fixed in 4% formaldehyde after 4 h. Fixed cells were permeabilized, processed for click chemistry as described above, and labeled with mouse monoclonal antibody 1A5 labeled with goat anti-mouse Alexa Fluor 568 (Thermo Fisher Scientific), rabbit anti-Cp labeled with goat anti-rabbit Alexa Fluor 647 (Thermo Fisher Scientific), and Hoechst nuclear stain. Coverslips were mounted on glass slides using ProLong glass antifade mountant (Thermo Fisher Scientific) and sealed with nail polish. The number of parasites at each life stage was counted from 10 fields using a 100× oil immersion objective on a Zeiss Axioskop Mot Plus fluorescence microscope, and the sum was divided by the total number of C. parvum parasites for that time point. Ratios were averaged across three independent experiments per compound (six total independent experiments for the DMSO control).

### Macrogamont inhibition assay.

HCT-8 cells were plated on 12-mm-diameter glass coverslips (Thermo Fisher Scientific) in 24-well tissue culture plates and incubated until confluency (∼24 h). Monolayers were infected with unfiltered, excysted oocysts (0.75% sodium taurocholate for 1 h at 37°C), washed twice with DPBS at 4 hpi, and cultured in fresh medium in the absence of compound. At 36 hpi, medium was replaced with compounds at their EC_90_s (Table 1) in HCT-8 medium containing 1% DMSO and incubated for an additional 36 h before fixation at 72 hpi with 4% formaldehyde. Coverslips were labeled with mouse monoclonal antibody 4D8 labeled with goat anti-mouse Alexa Fluor 488 (Thermo Fisher Scientific), rabbit anti-Cp labeled with goat anti-rabbit Alexa Fluor 568 (Thermo Fisher Scientific), and Hoechst stain. The ratio of 4D8^+^ parasites to the total number of C. parvum cells was counted for 10 fields of view on a Zeiss Axioskop Mot Plus fluorescence microscope with a 100× oil immersion objective, and the percent inhibition of 4D8^+^ macrogamonts was calculated for each compound relative to the DMSO control for that experiment. The data represent three independent experiments per compound.

### Stage-specific antibody labeling and confocal microscopy.

HCT-8 cells were plated on 12-mm-diameter glass coverslips (Thermo Fisher Scientific) in 24-well tissue culture plates and incubated until confluency (∼24 h). Monolayers were infected with excysted sporozoites, washed twice with DPBS at 4 hpi, and treated with compounds at their EC_90_s (Table 1) in HCT-8 medium containing 1% DMSO. Coverslips were fixed in 4% formaldehyde for 10 min, permeabilized and blocked in PBS plus 1% BSA and 0.1% Triton-X, and labeled with primary antibodies, namely, mouse monoclonal antibody 1E12, 5E3, or 1B5, and either rabbit anti-Cp or anti-RH. Mouse monoclonal antibodies were labeled with goat anti-mouse Alexa Fluor 488 (Thermo Fisher Scientific), and rabbit polyclonals were labeled with goat anti-rabbit Alexa Fluor 647 (Thermo Fisher Scientific). DNA was stained with Hoechst.

Epifluorescence images were acquired on a Zeiss Axioskop Mot Plus fluorescence microscope with a 100×, 1.4 numerical aperture (NA) Zeiss Plan-Apochromat oil objective and an AxioCam MRm monochrome digital camera. Images were acquired using AxioVision software (Carl Zeiss, Inc.) and manipulated in ImageJ (https://fiji.sc/). Confocal z-stacks were acquired on a Zeiss LSM880 confocal laser scanning microscope with a 63×, 1.4 NA Zeiss Plan-Apochromat oil objective and Airyscan processing using the ZEN 2.1 black edition software. Three-dimensional images were generated using the visualization module of Volocity version 6.3 (Improvision).

### Transmission electron microscopy and measurement of feeder organelle area.

HCT-8 cells were plated in 6-well culture plates and incubated for ∼24 h until confluency. Monolayers were infected with excysted sporozoites, washed twice with DPBS at 4 hpi, and treated with compounds at their EC_90_s in HCT-8 medium containing 1% DMSO. At 12 hpi, cell monolayers were scraped, pelleted, and fixed for electron microscopy in 2% paraformaldehyde–2.5% glutaraldehyde (Polysciences, Inc., Warrington, PA) in 100 mM sodium cacodylate buffer (pH 7.2) for 2 h at room temperature and then overnight at 4°C. Samples were washed in sodium cacodylate buffer at room temperature and postfixed in 1% osmium tetroxide (Polysciences, Inc.) for 1 h. Samples were then rinsed extensively in distilled water (dH_2_O) prior to *en bloc* staining with 1% aqueous uranyl acetate (Ted Pella, Inc., Redding, CA) for 1 h. Following several rinses in dH_2_O, samples were dehydrated in a graded series of ethanol and embedded in Eponate 12 resin (Ted Pella, Inc.). Sections of 95 nm were cut with a Leica Ultracut UCT ultramicrotome (Leica Microsystems, Inc., Bannockburn, IL), stained with uranyl acetate and lead citrate, and viewed on a 1200 EX transmission electron microscope (JEOL USA, Inc., Peabody, MA) equipped with an AMT 8 megapixel digital camera and AMT Image Capture Engine V602 software (Advanced Microscopy Techniques, Woburn, MA). To capture parasites with similar orientations between treatment groups, single-nucleus trophozoites were only imaged if a sizeable host-parasite interface with an electron dense pedestal was present. Feeder organelles were manually outlined in each image, and the surface area within the outline was calculated in ImageJ.

### Quantification and statistical analyses.

All statistical analyses were performed in Prism (GraphPad). A two-way ANOVA corrected for multiple comparisons by Sidak’s method was used to compare compound treatments in ALI. For treatments in HCT-8 cells, data were analyzed with a one-way ANOVA (after confirming normality with a Shapiro-Wilk test), followed by Dunnett’s multiple-comparison test. For the macrogamont inhibition assay, data were analyzed with a nonparametric Kruskal-Wallis test, followed by Dunn’s test for multiple comparisons. Data on the area of feeder organelles were analyzed with a parametric two-tailed, unpaired Student *t* test after confirming normality with a Shapiro-Wilk test.
