# Supplementary figures and images for: Defining Stage-Specific Activity of Potent New Inhibitors of Cryptosporidium parvum Growth In Vitro
Source: mBio. 2020 Mar 3;11(2):e00052-20. doi: 10.1128/mBio.00052-20 (PMC7064746; doi:10.1128/mBio.00052-20)

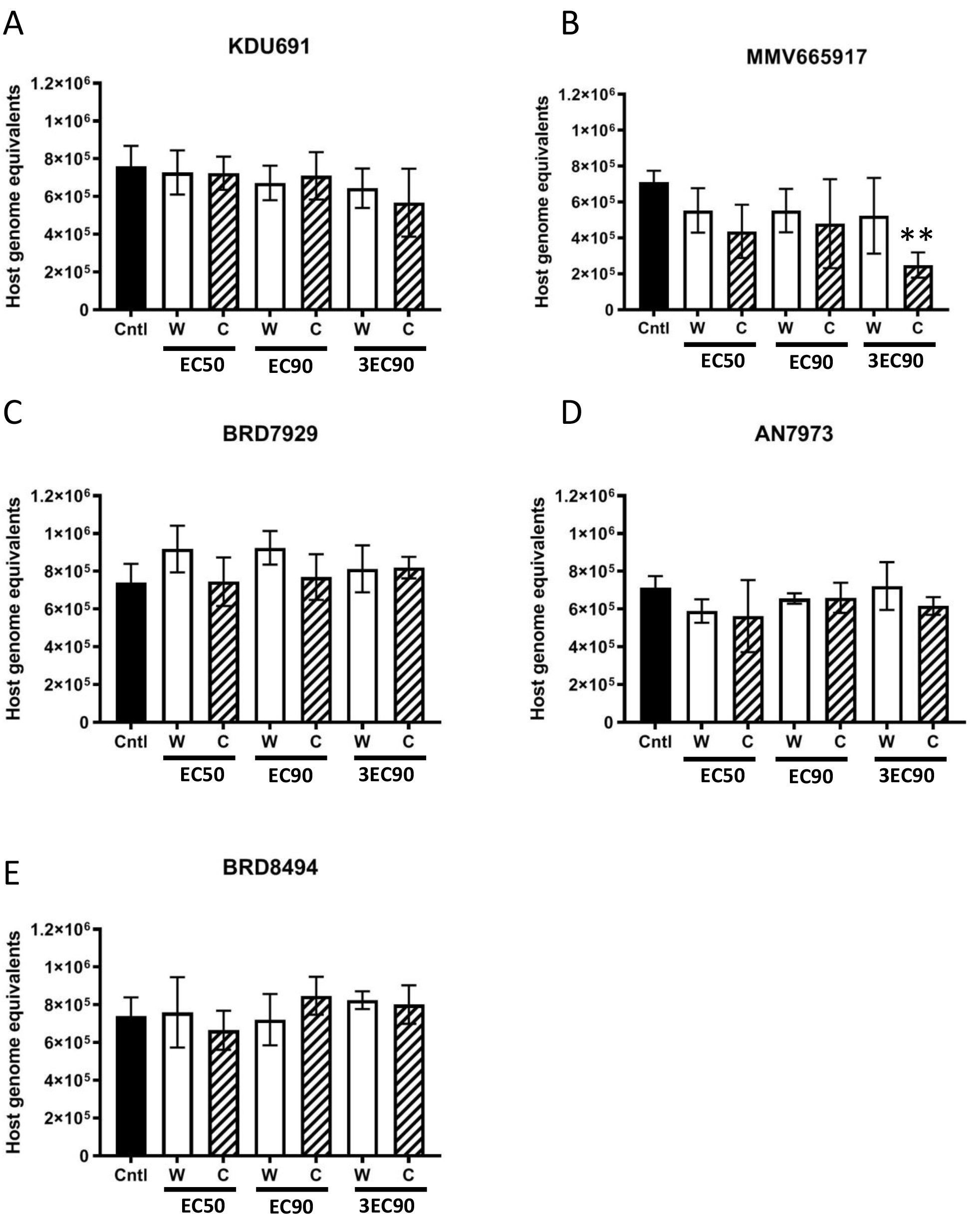

Supplement: FIG S1 [file mBio.00052-20-sf001.tif]

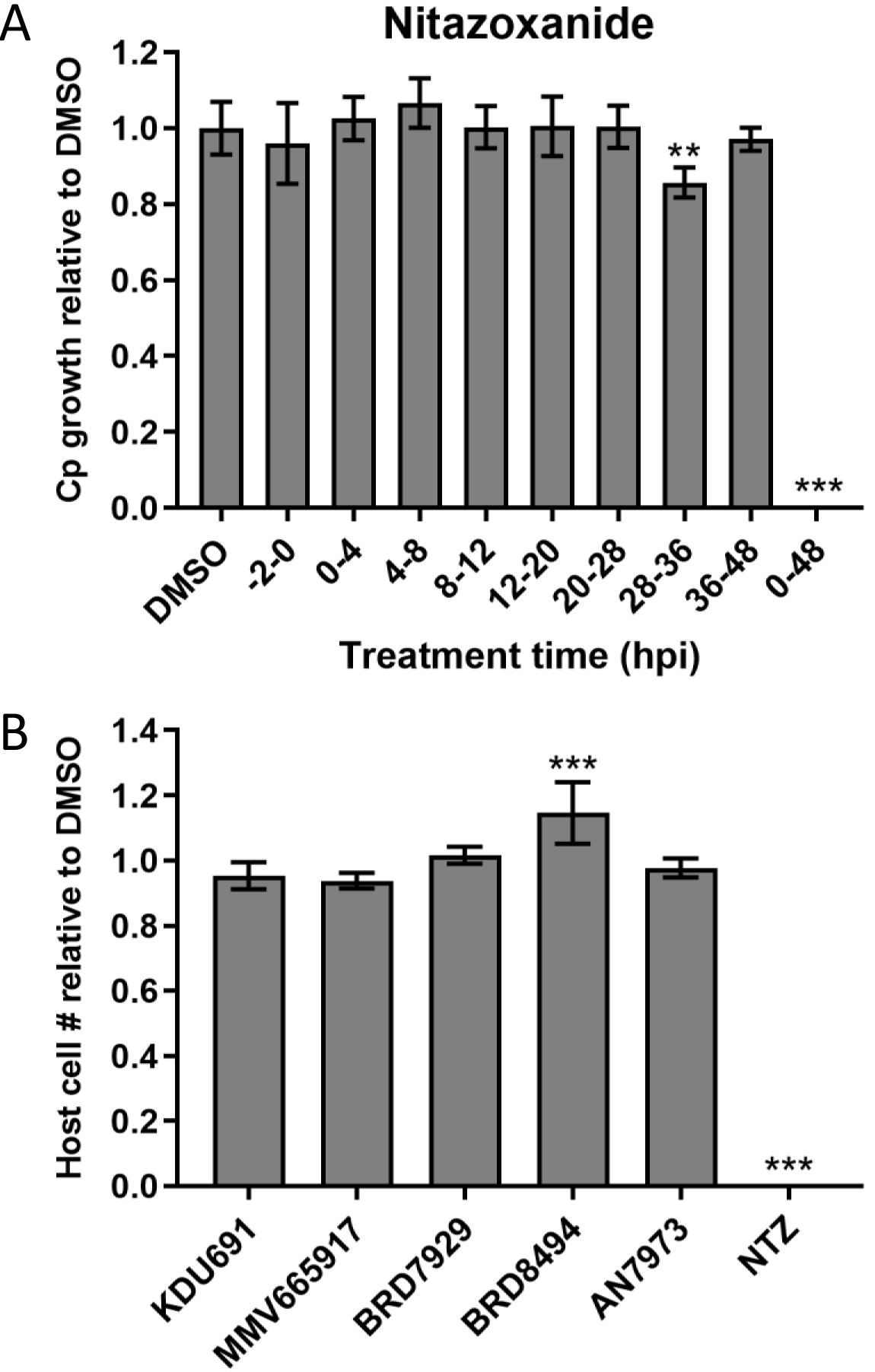

Supplement: FIG S2 [file mBio.00052-20-sf002.tif]

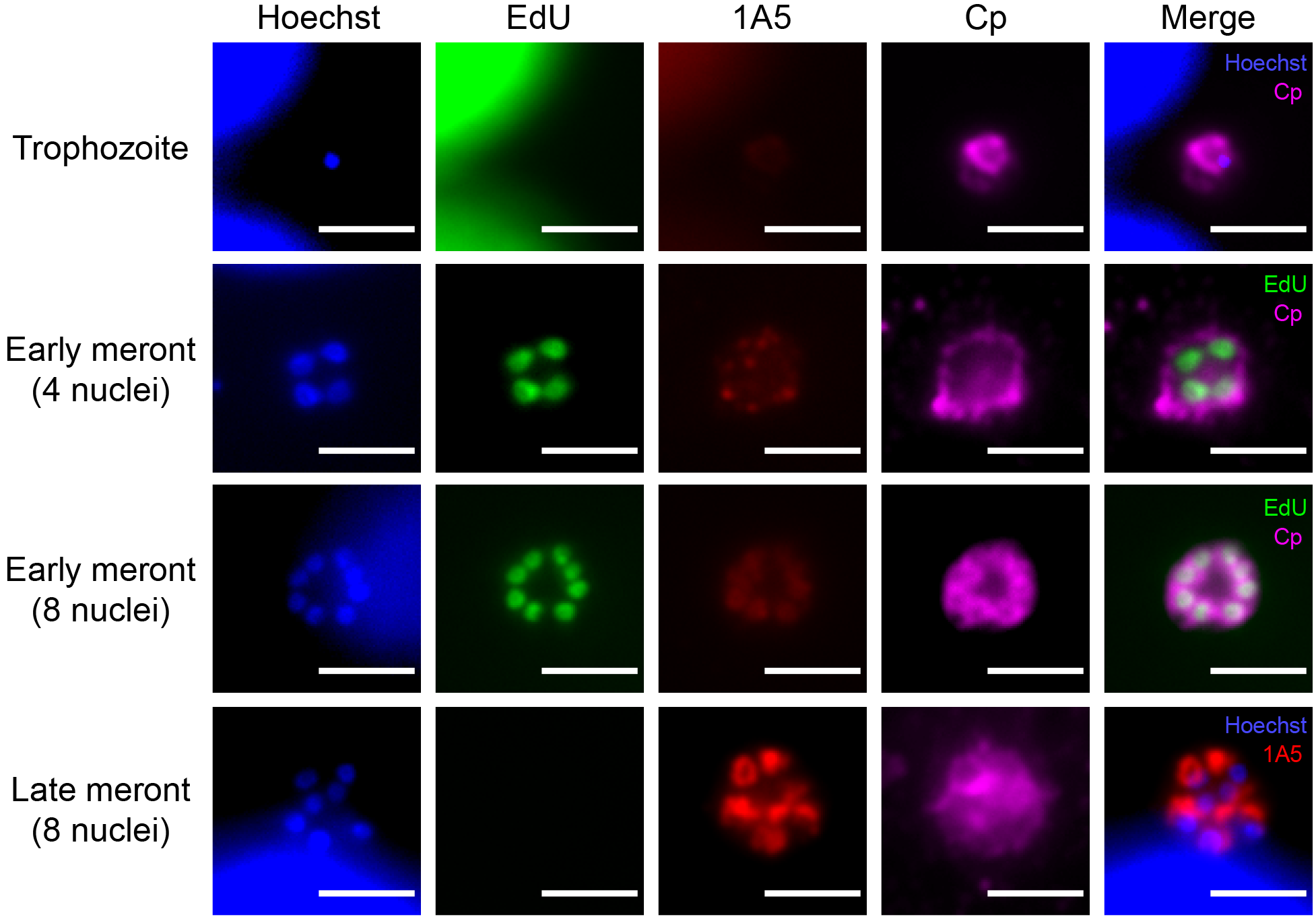

Supplement: FIG S3 [file mBio.00052-20-sf003.tif]

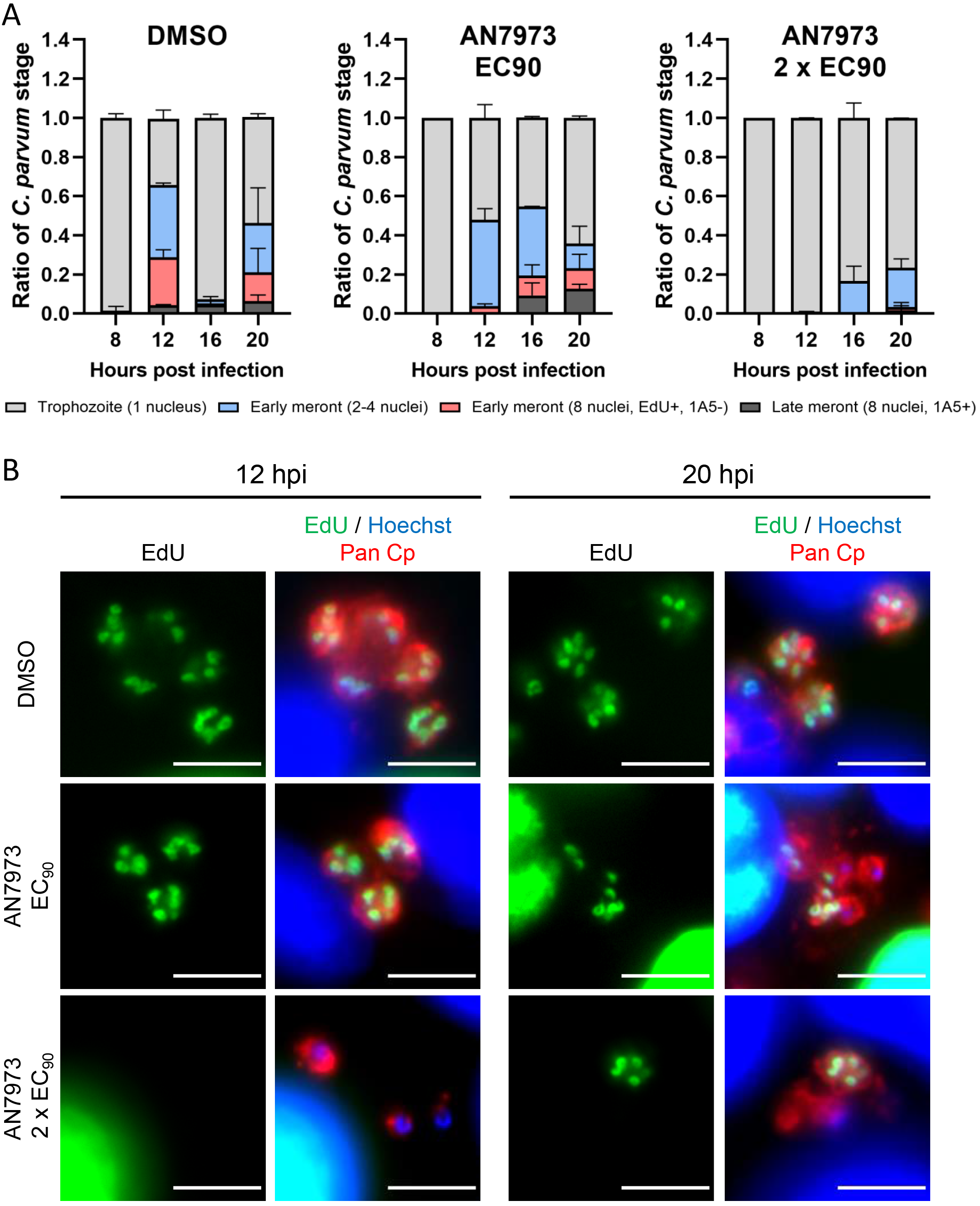

Supplement: FIG S4 [file mBio.00052-20-sf004.tif]

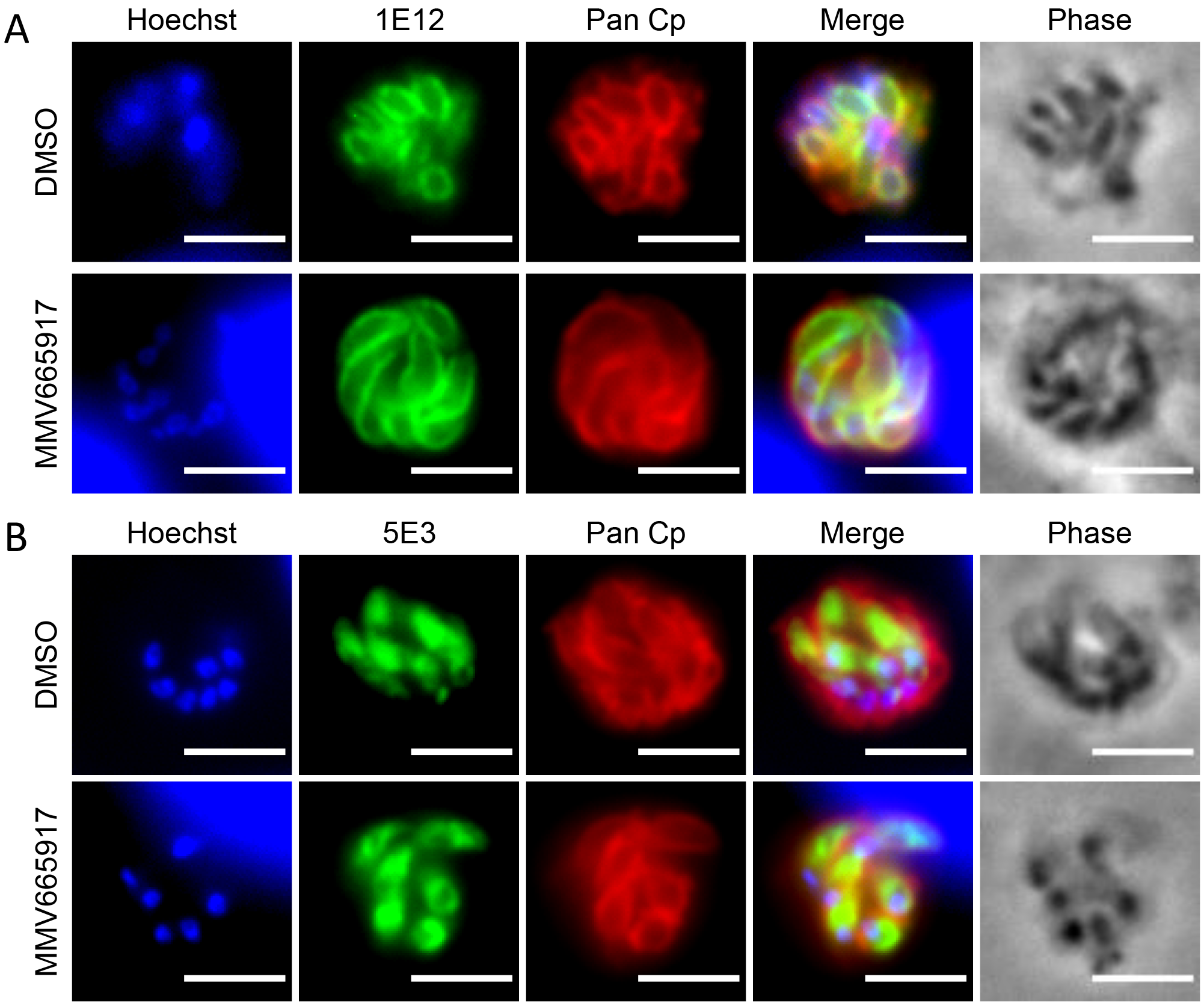

Supplement: FIG S5 [file mBio.00052-20-sf005.tif]
